# Clinical Outcomes of Dialysis-Treated Acute Kidney Injury Patients at the University of Port Harcourt Teaching Hospital, Nigeria

**DOI:** 10.5402/2013/540526

**Published:** 2012-09-05

**Authors:** Pedro Chimezie Emem-Chioma, Datonye Dennis Alasia, Friday Samuel Wokoma

**Affiliations:** Renal Unit, Department of Internal Medicine, University of Port Harcourt Teaching Hospital, PMB 6173, Rivers State, Port Harcourt 50001, Nigeria

## Abstract

*Background.* Acute kidney injury in adults is a common cause of hospitalization, associated with high morbidity and mortality especially in developing countries. In spite of RRT the in-hospital mortality rates remain high even in the developed countries. Though a proportion of our patients receive renal replacement therapy as part of their management, data on outcomes are sparse. *Study Objective.* To determine the clinical outcomes of dialysis-treated AKI in our hospital. *Methods.* A retrospective analysis of the clinical data of all adult AKI patients treated with haemodialysis at the University of Teaching Hospital during an interrupted six-year period was conducted. Analysis was done using SPSS version 17.0. *Results.* 34 males and 28 females with mean age of 41.3 ± 18.5 years were studied. The leading causes of AKI were sepsis (22.7%), acute glomerulonephritis (20.5%), acute gastroenteritis (15.9%), and toxic nephropathies (11.4%) and presented with mean e-GFR of 14.7 ± 5.8 mls/min/1.73 m^2^. Of the 62 patients, 29 (46.8%) were discharged from the hospital, 27 (43.5%) died in hospital, while 6 (9.7%) absconded from treatment. Survivors had better Rifle grade than those who died (*P* < 0.001). *Conclusion.* Hospital mortality rate of dialysis-treated AKI patients is high and the severity of renal damage at presentation may be an important factor.

## 1. Introduction

 Acute kidney injury (AKI) in adults is a common cause of hospitalization, associated with high morbidity and mortality especially in developing countries. Whereas community acquired AKI is more prevalent in the developing countries, hospital-acquired AKI is more prevalent in developed countries [1, 2]. Community-acquired AKI is responsible for about 1.4–1.9 percent of medical admissions in most series in Nigeria [3–5], while hospital-acquired AKI is responsible for about 1 percent of hospital admission most countries in Europe and North America [1, 2].

In view of the high morbidity and mortality associated with AKI, irrespective of type, there is an increasing application of renal replacement therapy (RRT) as a modality of treatment for AKI in the developed and developing countries. In spite of RRT interventions in the form of intermittent haemodialysis (IHD) or continuous therapies (CRRT), in- hospital mortality rates remain high in the developed countries of Europe and North America [6, 7]. Recent studies also indicate a rather high prevalence of progression to chronic kidney disease and ends stage kidney failure among survivors of RRT-treated AKI [8, 9]. This has been attributed to presence of the multiple organ dysfunctions in the predominantly ICU-based populations of AKI in those populations.

In Nigeria with a predominance of community acquired AKI, there is a relative paucity of systematic studies of RRT treated AKI patients in terms of overall renal and patient outcomes. Available information can only be obtained from inferences from data from studies of acute renal failure.

We under took a retrospective evaluation of the dialysis performance and outcomes of IHD-treated AKI patients in our hospital during an interrupted period of six years to determine the prevalence of haemodialysis-treated AKI and to determine the haemodialysis exposure and the overall outcome of haemodialysis-treated AKI in adult patients in our center. 

## 2. Methods

A retrospective analysis of the clinical data of all adult AKI patients treated with IHD during an interrupted six year-period. The history of haemodialysis service at the university of Port Harcourt Teaching Hospital falls into two operational periods. The first period was from July 1996 to December 1999 when operations ceased due to technical problems. In January 2007, operations recommenced following installation of four haemodialysis machines. 

The clinical case files of all haemodialysis-treated acute kidney injury (AKI) patients during the two periods: July 1996 to December 1999, (a period of three and half years) and January 2007 to June 2009 (two and half years), making a total of six years, were obtained for analysis. 

Data for analysis obtained from the haemodialysis case records and the in-patient case files, include the demographic data, the major clinical features at presentation, the aetio-pathologic cause of AKI as documented by the managing renal team, the daily urine output record, the haematocrit level at presentation, the electrolytes, urea and creatinine profiles at presentation and subsequently for the period of hospitalization, as well as the indication(s) for haemodialysis.

The haemodialysis data obtained include the following: (1) measures of dialysis exposure; the duration of haemodialysis therapy and the total number of dialysis sessions attained, before the study end point. Intermittent haemodialysis was the only form of renal replacement therapy available in our center. All the 62 patients received IHD. (2) Measures of renal and clinical outcomes: these include 24-hr urine output profile, change in the serum urea and creatinine levels, progression of e-GFR, and the overall patient clinical recovery.

Study end points include, discharge from hospital, dialysis dependence beyond 12 weeks, abscondment from treatment or death. 

The e-GFR of the patients was calculated using the Cockcroft and Gault [10] equations for males and females.

Acute kidney injury was defined in accordance with Acute Dialysis Quality Initiative (ADQI) guidelines [11] as a sudden deterioration in kidney function characterized by oligo-anuria or normal urine volume, rapid rise in plasma levels of urea, and creatinine and rapid fall in the e-GFR occurring within a few days and usually lasting no longer than 12 weeks. The severity of AKI at presentation was assessed using the RIFLE criteria [11]. Because the urine output records as obtained in the case records were found not likely to be accurate, the serum creatinine level (Scr) in mg/dL was used for the determination of the Rifle grade of the patients. 

The premorbid Scr levels of the patients were not known as they all presented for the first time in AKI (community-acquired AKI). In accordance with the Acute Kidney Injury Network (AKIN) [11] recommendation, the estimated pre-morbid Scr of the patients were calculated using the modification of diet in renal disease (MDRD) formula for determination of e-GFR, with an assumed e-GFR of 75 mls/min/1.73 m^2^.

In accordance with the *Rifle* grading using Scr, *Risk* = Scr 1.5 fold of baseline, *Injury* = Scr 2 fold of baseline, *Failure* = Scr 3 fold of baseline or Scr ≥ 4 mg/dL, *Loss* = persistent ARF or complete loss of kidney function, and *End* stage is kidney failure, lasting more than 3 months.

## 3. Data Management 

The data analysis was done using SPSS version 17.0. Quantitative data are presented as mean ± s.d, and categorical variables as percentages. Statistical comparisons were done using independent sample *t*-test and Chi-square test as appropriate, while Pearson's correlation test was used to establish relationship between variables. Significant levels were set at *P* < 0.05.

## 4. Results 

During the period understudy a total of 6151 medical admissions were recorded, of which 735 (11.9%) were cases of kidney failure, comprising 614 (9.9%) chronic kidney failure, and 121 (1.9%) cases of acute kidney injury (AKI). 

 Out of the 121 cases of AKI, 62 (51.2%) received renal replacement therapy in the form of intermittent haemodialysis as part of their management. The hospital prevalence of dialysis-treated AKI was therefore 1.0% of medical admissions, 8.4% of all kidney failure cases, and 51.2% of AKI patients. 

The clinical data and the outcomes of the 62 patients who received renal replacement therapy in the form of acute intermittent haemodialysis were further analyzed.

 They comprised 34 males and 28 females (M/F = 1.2 : 1) with a mean age of 41.3 ± 18.5 (15–83) years. The peak age was in the 40–49 age-group. Over 70% of the patients were under fifty years of age. The baseline parameters of the patients at entry are set out in Table 1.

The clinical settings for AKI were medical 44 (70.9%), surgical 15 (24.2%), and obstetrics 3 (4.8%), respectively. The leading medical aetio-pathologies causing AKI were, sepsis (22.7%), acute glomerulonephritis 9 (20.5%), acute gastroenteritis 7 (15.9%), and toxic nephropathies 5 (11.4%), respectively.

Obstructive uropathy 9 (60.0%) was the commonest surgical cause of AKI, while obstetric haemorrhages 2 (66.7%) were the commonest obstetric causes of AKI. 

The four most frequent presenting features (Table 2) were oligo-anuria 37 (59.7%), oedema 30 (48.4%), encephalopathy 17 (27.4%), and fever 8 (13.3%).

Their mean systolic and diastolic blood pressures at presentation were 150.5 ± 26.5 (100–220) mmHg and 92.5 ± 19.5 (60–160) mmHg, respectively. Hypertension (BP ≥ 140/90 mmHg) was observed in 34 (54.8%) of the patients. 

The indications for dialysis in the patients were very high azotaemia at presentation or rapidly rising azotaemia 39 (48.4%), severe metabolic acidosis (serum bicarbonate levels < 10 mmol/L) 19 (30.7%), uraemic encephalopathy 20 (32.3%), prolonged anuria 3 (4.8%) acute pulmonary oedema, and severe hyperkalemia (plasma potassium ≥ 6.5 mmol/L) 3 (3.0%) each, respectively. 

The mean e-GFR of the patients at presentation was quite low with a mean of 14.7 ± 5.8 (6.7–34) mls/min/1.73 m^2^.

The estimated mean pre-morbid serum creatinine was 1.27 ± 0.33 mg/dL (0.73–2.15). 

The mean of the ratio of the actual serum creatinine at presentation to the estimated pre-morbid serum creatinine was 8.55 ± 3.9 (2.15–17.6) folds.

Applying the Rifle grading for AKI, based on serum creatinine levels at presentation, the results were *Risk* (0.0%), *Injury* 4 (6.5), *Failure* 58 (93.5%), *Loss* and *End* stage kidney failure could not be evaluated. 

## 5. Dialysis Exposure and Clinical Outcomes

The mean number of dialysis sessions received was 2.3 ± 1.7, with a range of 1 to 8 sessions.

**Figure 1:**
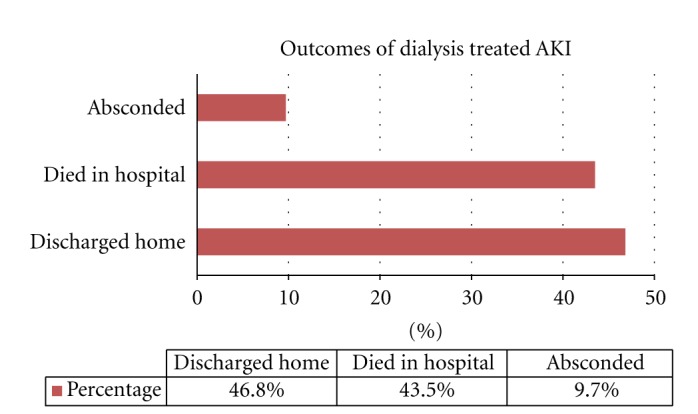
Outcomes of dialysis-treated AKI.

Of the 62 patients, 29 (46.8%) recovered enough to be discharged from the hospital, 27 (43.5%) died in hospital while 6 (9.7%) absconded from treatment (Figure 1). 

There was no significant difference between the number that survived and the number that died (*P* > 0.05).

 Comparison between the demographic, clinical, and Rifle status of the dead and the surviving patients (Table 3) showed that the surviving patients had a better Rifle grade than those that died. All the mortality cases had 100 percent Rifle-failure status compared to 85 percent among the survivors (*P* < 0.001). 

## 6. Discussions

The results from our study show that dialysis-treated AKI is quite prevalent in our hospital, accounting for one percent of medical admissions, 8.4 percent of kidney failure cases, and 51.2 percent of acute kidney injury patients. Thus over fifty percent of our acute kidney failure patients received RRT. 

The percentage of AKI patients requiring dialysis may be higher as some may have needed dialysis but did not have access due to the high cost of haemodialysis in Nigeria [12].

Though published studies in Nigeria focusing on dialysis-treated AKI is sparse, inferences from the data on studies of acute renal failure in Nigerian patients indicate that about 29.2–90.6 percent of acute kidney injury patients received dialysis in the course of their treatment [3–5].

Data from studies from other developing countries show prevalence rates similar to ours. Jayakumar et al. [13] in a study of epidemiological trend in acute renal failure in South Indian region recorded 69% dialysis requiring AKI. Similarly, Hassan et al. [14] in Pakistan recorded 100 percent dialysis treatment.

 In the developed countries of Europe and North America, hospital-acquired AKI dominates over community-acquired AKI, being predominantly from postoperative (major surgeries such as cardiac transplant) sepsis and haemodynamic instabilities from multiorgan dysfunction. Most cases are intensive care unit (ICU) based. In the United states of America hospital acquired AKI accounts for 1 percent of hospital admissions while in the United Kingdom it is responsible for 175–200 per million population per year [6, 7]. In these populations it is estimated that 4-5 percent of general intensive care unit populations would require RRT [15].

 In these settings, RRT requirement and therapy is quite prevalent and increasingly becoming the gold standard of care for AKI patients [16, 17].

 From various reports a wide range of between 20 and over 80 percent of ICU based AKI patients are treated with one form of RRT or the other and population estimates indicate that RRT-requiring AKI occur in 200–300 per million population per year [18, 19]^.^ The clinical and metabolic profiles of our patients were characterized by poor status with severe azotemia, metabolic acidosis, persistent oliguria, and uraemic encephalopathy (Tables 1, 2 and 3). Their mean entry serum creatinine (11.6 ± 5.4 mg/dL) was several multiples (11.3 folds) of their estimated mean pre-morbid Scr (1.24 mg/dL). Their entry mean e-GFR were quite low (14.7 ± 5.6 mls/min/1.73 m^2^) and their Rifle status at entry were also severe, with 93.5% in the failure category. The patients were extremely ill; hence the 51.2 percent need for RRT intervention and the 43.5% mortality recorded was not surprising. Though, measures of severity of illness like APACHE II, SAPS II, and SOFA scores were not documented for the patients. APACHE II score has been shown to reproducibly predict mortality in patients who are critically ill, including critically ill patients with AKI [20].

Factors responsible for their poor clinical and metabolic state would include late presentation in advanced stage of AKI, predominantly community acquired nature of AKI, whose precipitating ailments were predominantly, sepsis, entero-invasive acute gastroenteritis, toxic nephropathies, as previously documented in AKI in developing countries [21, 22]. These causes of AKI are known to inflict severe renal injury necessitating RRT therapy [6, 7] and often associated with poor renal and patient outcomes.

We recorded 43.9 percent mortality among RRT-treated AKI patients (Figure 1). Though there are no similar statistics of mortality rates among RRT-treated Nigerians, the overall AKI mortality rates are high in Nigeria. Recent data from Benin, Ilorin, Ile-Ife, all in Nigeria showed 36%, 62%, and 38.7% mortalities in all AKI patients, respectively [3–5].

 Our mortality figures are similar to in-hospital mortality figures of RRT-treated patients in developed centers, (with better facilities such as continuous renal replacement therapies-CRRT), which range from 30 to 80 percent [23, 24]. However, there seems to be no difference in outcome between the use of IHD and CRRT, though CRRT may have a role in patients who are haemodynamically unstable and who had prolonged renal failure after a stroke or liver failure. The Acute Renal Failure Trial Network (ATN) study found that no additional benefit (morbidity or mortality) was conferred to patients who received more intensive dialysis (either intermittent or continuous dialysis) compared to those who received less intensive dose of RRT [25].

AKI serious enough to require RRT has been found to be associated with worse Rifle grade at presentation, high in-hospital mortality and progression to chronic kidney disease (CKD) and end-stage kidney disease (ESRD) in 5–20 percent of survivors within a few years [8]. A US-based multicenter analysis of data among US Veteran Affairs population, followed up for over 60 months after episodes of RRT-requiring AKI, showed that AKI increased the risk of developing CKD-4 by 303 to 550 percent [9]. All the patients who developed CKD did so within 20 months of discharge from the hospital. This emphasizes the need to keep AKI survivors under close medical surveillance over a long period of time. Over 93 percent of our patients had failure-grade of Rifle at presentation and associated high in-hospital mortality. Due to poor follow up it could not be ascertained what proportion of the survivors developed CKD after discharge from hospital.

The poor dialysis exposure of our patients in terms of dialysis frequency and total number of sessions received may have contributed to the high in-hospital mortality. 

The most dialyzed patient achieved 8 dialysis sessions over a period of 6.5 weeks. The apparent low frequency of dialysis was due to poor financial status of the patients and frequency of dialysis was similar in both survivors and those that died (Table 4). Access to dialysis therapy remains a serious challenge in resource poor developing countries like Nigeria [26, 27]. This is mainly due to poor access resulting from the relative high cost of dialysis treatment [11].

Comparison between the survivors and those that died in our series showed that there were no statistically significant differences in the demographic and metabolic characteristics. All the patients were managed on the general wards and there were records of treatment with ionotropes or mechanical ventilation in these patients. The survivors however had a statistically significant lower percentage of the Rifle-failure grade, 85% versus 100%, respectively, (*P* < 0.001) (Table 4). This is in keeping with previous observation demonstrating the usefulness of the Rifle grade in prognostication in AKI. AKI patients presenting in the *Risk* and *Injury* grade have better outcomes than those presenting in the advanced grades of *Failure*, *Loss*, or *End* stage categories [28, 29].

Given the rather poor outcome of dialysis-requiring AKI patients both in the developing and developed countries, there is the need for preventive measures to reduce the prevalence of AKI in the community and in the hospital.

Though hospital acquired AKI has not been systematically studied in Nigeria, its incidence and prevalence are likely to be high. Accident and emergency units, burns units, children emergency units, obstetrics units, postoperative surgical wards, and the few emerging ICUs in the country are focal points for hospital acquired AKI. All patients admitted in these settings are at high risk of developing AKI.

Medical care givers working in these areas need to be more proactive and anticipatory in their approach to patients. All patients admitted into these facilities should have urine tests performed, urine volumes, and fluid balance charts properly kept. Serial plasma urea, creatinine, and electrolytes tests should be done upon admission and frequently thereafter. In this way impairments in renal function can be detected early and early intervention given. Patient's state of hydration, blood or fluid loss and early evidence of sepsis should be proactively anticipated and early interventions undertaken. In this way cases of prerenal renal injuries, which progress to AKI, can be prevented.

 Unfortunately, these are usually not done, it is when a patient becomes oliguric or anuric that attention is paid to the kidneys. Quite often, renal consults are not made until patient's clinical state becomes critical, requiring emergency RRT.

Prevention of community-acquired AKI is more difficult as most patients present when AKI is fully established. However the common causes of community-acquired AKI are communicable diseases or exposure to environmental toxins, for which prevention is possible. Malaria, cholera, snake bites, entero-invasive gastroenteritides, toxic nephropathies, and so forth are all potentially preventable by appropriate public health interventions. It is through such public health interventional measures that community acquired AKI have been virtually eliminated in most developed countries of Europe and North America [6, 7]. The challenge is thus before our Public health departments at the state and National levels.

 AKI progressing to CKD and ESRD possibly contribute to the high prevalence of CKD in our populations. A proportion of the 3–5 percent of the indeterminate causes of CKD in our environment [30, 31] may be due to partially resolved AKI which gradually progress to CKD over time. Follow up of discharged AKI patients is relatively poor in Nigeria. This is due to a number of factors ranging from high absconding rates, ignorance, poor communication facilities, long distances, and difficult terrains between city-based hospitals and the patients' rural domains, and so forth. With the advent of the mobile phone communication system, which is widely subscribed to, even amongst poor and rural based people in Nigeria, there is need for medical care givers and our medical social services units to be more proactive in reaching patients discharged from hospital to improve follow up of discharged patients. Patients who failed to turn up on their appointed follow-up days can be reached on phone. 

With improved follow ups we would be able to track our surviving post-AKI patients to determine their long term outcomes.

## 7. Conclusions

The results from this study show that the prevalence of dialysis-treated AKI is high in our hospital. The patients present in advanced Stages of Rifle grading with high in-hospital mortality rates. Late presentation, advanced Rifle stage at presentation and inadequate dialysis exposure account for the high in-hospital mortality rates observed.

## 8. Study Limitations

Being a retrospective study, data retrieval may have been deficient. The haemodialysis case files of all 62 patients were however retrieved as they were stored in the haemodialysis unit. This can however not be said for the clinical case files which are kept in the hospital medical records library.

The fluid charts as recorded in the in-patient case files were considered unreliable. They were thus not used in the definition and Rifle grading of AKI in the patients.

## Figures and Tables

**Table 1:** Baseline clinical parameters of AKI patients.

Parameters	Mean ± SD; %
Age (years)	41.3.0 ± 18.75
Sex (M/F)	38/24 (1.6:1.)
Clinical Setting	
Medical	44 (70.9%)
Surgical	15 (24.2%)
Obstetrics	3 (4.8%)
Rifle grade	
Risk	Nil
Injury	4 (6.5%)
Failure	58 (93.5%)
Loss	—
End stage	—

**Table 2:** Baseline laboratory parameters of AKI patients.

Parameters	Mean ± SD
Haematocrit (%)	25.4 ± 6.9
Sodium (mmol/L)	135.0 ± 9.1
Bicarbonate	15.3 ± 5.2
Urea (mmol/L)	31.65 ± 12.4
Creatinine (mg/dL)	11.6 ± 5.4
e-GFR (mls/min/1.73 m^2^)	14.7 ± 5.8
Estimated premorbid-Scr (mg/dL)	1.27 ± 0.3

**Table 3:** Clinical features at presentation.

Clinical features	Frequency	Percentage
Oligo-anuria	37	59.7
Oedema	30	48.4
Encephalopathy	17	27.42
Fever	8	13.3
Hiccup	4	6.5
Vomiting	4	6.5
Haemoptysis	3	4.8
Pulmonary oedema	3	4.8
Loin pains	1	1.6
Jaundice	1	1.6

**Table 4:** Comparison between survivors and mortalities.

Parameters	Survivors (*n* = 29)	Mortalities (*n* = 27)	*P* value
Age	42.1 + 18.6	43.9 ± 18.7	*P* > 0.05 (ns)
Sex ratio (M/F)	1.6 : 1	1.7 : 1	No difference
Haematocrit (%)	24.6 ± 5.9	26.1 ± 7.8	*P* > 0.05 (ns)
Sodium (mmol/L)	133.8 ± 9.1	136.2 ± 9.2	*P* > 0.05 (ns)
Potassium (mmol/L)	4.7 ± 1.1	4.6 ± 1.1	*P* > 0.05 (ns)
Bicarbonate (mmol/L)	15.24 ± 5.38	15.3 ± 5.1	*P* > 0.05 (ns)
Urea (mmol/L)	29.33 ± 14.21	33.4 ± 10.6	*P* > 0.05 (ns)
Creatinine (mg/dL)	11.62 ± 5.83	11.5 ± 4.9	*P* > 0.05 (ns)
e-GFR (mls/min/1.72 m^2^) (at entry)	14.21 ± 6.87	14.7 ± 5.6	*P* > 0.05 (ns)
Estimated premorbid scr (mg/dL)	1.26 ± 0.30	1.22 ± 2.15	*P* > 0.05 (ns)
Duration on dialysis (weeks)	2.5 ± 1.96	2.3 ± 1.4	*P* > 0.05 (ns)
No of dialysis sessions received	2.6 ± 1.7	2.3 ± 1.4	*P* > 0.05 (ns)
Rifle grade:			
Risk	Nil	Nil	
Injury	4 (14.8%)	Nil	
Failure	23 (85.2%)	27 (100%)	*P* < 0.001
Loss	—	—	
End stage kidney failure	—	—	

## References

[1] Schrier RW, Wang W, Poole B, Mitra A (2004). Acute renal failure: definitions, diagnosis, pathogenesis, and therapy. *Journal of Clinical Investigation*.

[2] Liaño F, Pascual J (1996). Epidemiology of acute renal failure: a prospective, multicenter, community-based study. *Kidney International*.

[3] Okoye OC, Unigbe EI, Ojogwu LI Acute kidney injury in adult Nigerians: a single center experience.

[4] Chijioke A, Aderibigbe A, Olarenwaju TO, Makusidi AM Pattern of Acute renal failure in Ilorin: a report.

[5] Udo AI, Arogundade FA, Sansui AA Acute kidney injury, a review of the causes, severity and outcome in Ile-Ife, Nigeria.

[6] Cerdá J, Bagga A, Kher V, Chakravarthi RM (2008). The contrasting characteristics of acute kidney injury in developed and developing countries. *Nature Clinical Practice Nephrology*.

[7] Lamier N, Bussan WV, Vanholder R (2006). The changing epidemiology of acute renal failure. *Nature Clinical Practice Nephrology*.

[8] Lo LJ, Go AS, Chertow GM (2009). Dialysis-requiring acute renal failure increases the risk of progressive chronic kidney disease. *Kidney International*.

[9] Andur RL, Chalwa LS, Arnodeo S, Kimmel PC, Palent CE (2009). Outcome following acute renal failure in United states veterans: focus on acute tubular necrosis. *Kidney International*.

[10] Cockcroft DW, Gault MH (1976). Prediction of creatinine clearance from serum creatinine. *Nephron*.

[11] Mehta RL, Kellum JA, Shah SV (2007). Acute kidney injury network: report of an initiative to improve outcomes in acute kidney injury. *Critical Care*.

[12] Unuigbe EI (2006). Funding Renal care in Nigeria, critical appraisal. *Tropical Journal of Nephrology*.

[13] Jayakumar M, Ram Prabahar M, Fernando E, Manorajan R, Venkatraman R, Balaraman V (2006). Epidemiologic trend changes in acute renal failure—a tertiary center experience from South India. *Renal Failure*.

[14] Hassan I, Junejo AM, Dawani ML (2009). Etiology and outcome of acute renal failure in pregnancy. *Journal of the College of Physicians and Surgeons Pakistan*.

[15] Hoste EAJ, Schurgers M (2008). Epidemiology of acute kidney injury: how big is the problem?. *Critical Care Medicine*.

[16] Saudan P, Niederberger M, De Seigneux S (2006). Adding a dialysis dose to continuous hemofiltration increases survival in patients with acute renal failure. *Kidney International*.

[17] Himmelfarb J (2007). Continuous dialysis is not superior to intermittent dialysis in acute kidney injury of the critically ill patient. *Nature Clinical Practice Nephrology*.

[18] Woodrow G, Turney JH (1992). Cause of death in acute renal failure. *Nephrology Dialysis Transplantation*.

[19] Metcalfe W, Simpson M, Khan IH (2002). Acute renal failure requiring renal replacement therapy: incidence and outcome. *QJM*.

[20] Wang IK, Wang ST, Chang HY (2005). Prognostic value of acute physiology and chronic health evaluation II and organ system failure in patients with acute renal failure requiring dialysis. *Renal Failure*.

[21] Bamgboye EL, Mabayoje MO, Odutola TA, Mabadeje AFB (1993). Acute renal failure at the Lagos University Teaching Hospital: a 10-year review. *Renal Failure*.

[22] Kohli HS, Bhat A, Jairam A (2007). Predictors of mortality in acute renal failure in a developing country: a prospective study. *Renal Failure*.

[23] Xue JL, Daniels F, Star RA (2006). Incidence and mortality of acute renal failure in Medicare beneficiaries, 1992 to 2001. *Journal of the American Society of Nephrology*.

[24] Schrier RW, Wang W (2004). Acute renal failure and sepsis. *The New England journal of medicine*.

[25] Palevsky PM, Zhang JH, O’Connor TZ (2008). Intensity of renal support in critically ill patients with acute kidney injury. *New England Journal of Medicine*.

[26] Arije A, Kadiri S, Akinkugbe OO (2000). The viability of hemodialysis as a treatment option for renal failure in a developing economy. *African Journal of Medicine and Medical Sciences*.

[27] Odutola TA, Ositelu SB, D’Almeida EA, Mabadeje AFB (1989). Five years experience of haemodialysis at the Lagos University Teaching Hospital - November 1981 to November 1986. *African Journal of Medicine and Medical Sciences*.

[28] Bellomo R (2005). Defining, quantifying, and classifying acute renal failure. *Critical Care Clinics*.

[29] Bell M, Liljestam E, Granath F, Fryckstedt J, Ekbom A, Martling CR (2005). Optimal follow-up time after continuous renal replacement therapy in actual renal failure patients stratified with the RIFLE criteria. *Nephrology Dialysis Transplantation*.

[30] Akinsola W, Odesanmi WO, Ogunniyi JO, Ladipo GOA (1989). Diseases causing chronic renal failure in Nigerians—a prospective study of 100 cases. *African Journal of Medicine and Medical Sciences*.

[31] Mabayoje MO, Bamgboye EL, Odutola TA, Mabadeje AFB (1992). Chronic renal failure at the Lagos University Teaching Hospital: a 10-year review. *Transplantation Proceedings*.

